# Polycyclic Aromatic Hydrocarbons (PAHs) in Indoor Dusts of Guizhou, Southwest of China: Status, Sources and Potential Human Health Risk

**DOI:** 10.1371/journal.pone.0118141

**Published:** 2015-02-26

**Authors:** Qin Yang, Huaguo Chen, Baizhan Li

**Affiliations:** 1 College of Urban Construction and Environmental Engineering, Chongqing University, Chongqing, P.R. China; 2 College of Civil Engineering, Guizhou University, Guiyang, Guizhou Province, P.R. China; 3 Engineering Laboratory for Quality Control and Evaluation Technology of Medicine, Guizhou Normal University, Guiyang, Guizhou Province, P.R. China; Peking University, CHINA

## Abstract

Polycyclic aromatic hydrocarbons (PAHs) were analyzed for 136 indoor dust samples collected from Guizhou province, southwest of China. The ∑18PAHs concentrations ranged from 2.18 μg•g-1 to 14.20 μg•g-1 with the mean value of 6.78 μg•g-1. The highest Σ18PAHs concentration was found in dust samples from orefields, followed by city, town and village. Moreover, the mean concentration of Σ18PAHs in indoor dust was at least 10% higher than that of outdoors. The 4–6 rings PAHs, contributing more than 70% of ∑18PAHs, were the dominant species. PAHs ratios, principal component analysis with multiple linear regression (PCA-MLR) and hierarchical clustering analysis (HCA) were applied to evaluate the possible sources. Two major origins of PAHs in indoor dust were identified as vehicle emissions and coal combustion. The mean incremental lifetime cancer risk (ILCR) due to human exposure to indoor dust PAHs in city, town, village and orefield of Guizhou province, China was 6.14×10^−6^, 5.00×10^−6^, 3.08×10^−6^, 6.02×10^−6^ for children and 5.92×10^−6^, 4.83×10^−6^, 2.97×10^−6^, 5.81×10^−6^ for adults, respectively.

## Introduction

Polycyclic aromatic hydrocarbons (PAHs) are chiefly byproducts of incomplete combustion of fossil fuels and biomass and pyrosynthesis of organic materials [1, 2]. PAHs are ubiquitous environmental pollutants that have been identified worldwide in various matrices, such as dust particle, water or soil, and include more than 100 kinds of PAH compounds. In view of their widespread sources and strong carcinogenicity, PAHs have been brought into extensive public attention and attracted greatly interest of experts and government organizations [3–5]. For example, the U.S. Occupational Safety and the Health Administration (OSHA) and the National Institute for Occupational Safety and Health (NIOSH) have announced exposure limit for PAHs content, and the American Conference of Governmental Industrial Hygienists (ACGIH) has established 46 biological exposure indices for over 100 chemical exposures including PAHs [6].

People spend more than 80% of their time indoors, and the research on indoor environment has gained more attentions. In recent decades, environmental contaminants including asbestos, heavy metals, pesticides, phthalates, and polychlorinated biphenyls have been investigated in indoor dust [7, 8]. PAHs, another important group of environmental contaminants, have been widely detected in soil, industrial effluent, marine bottom sediments, air, meat and seafood. Little research has been conducted, however, to evaluate the PAHs contamination in dust, especially in indoor dust which can easily become the carrier of pollutants, directly or indirectly by human inhalation or ingestion, and induce a variety of diseases [9].

In China, increasing anthropogenic emissions from rapid industrialization and urbanization have contributed to the serious PAHs pollution in some densely populated cities [10, 11]. Guizhou, located in the southwest of China, is a developing province with a total area of 176167 km^2^. In recently years, the local government has continuously devoted to the economic construction by greatly developing industry, but the following environment challenges would be conceivably more distinct [12–14]. The soils in Guiyang city, the largest city of Guizhou province, has been contaminated by PAHs at a medium level [15]. The exposure through ingestion and/or inhalation of indoor dust may be comparable to corresponding food consumption, especially for younger children [16]. However, PAHs contamination in indoor dust and the associated potential risk has not been investigated in West China. Therefore, the purpose of present research was to investigate the levels, distributions and possible sources of PAHs in indoor dust, and to further evaluate their potential health risks.

## Materials and Methods

### Dust sampling and preparation

**Fig 1:**
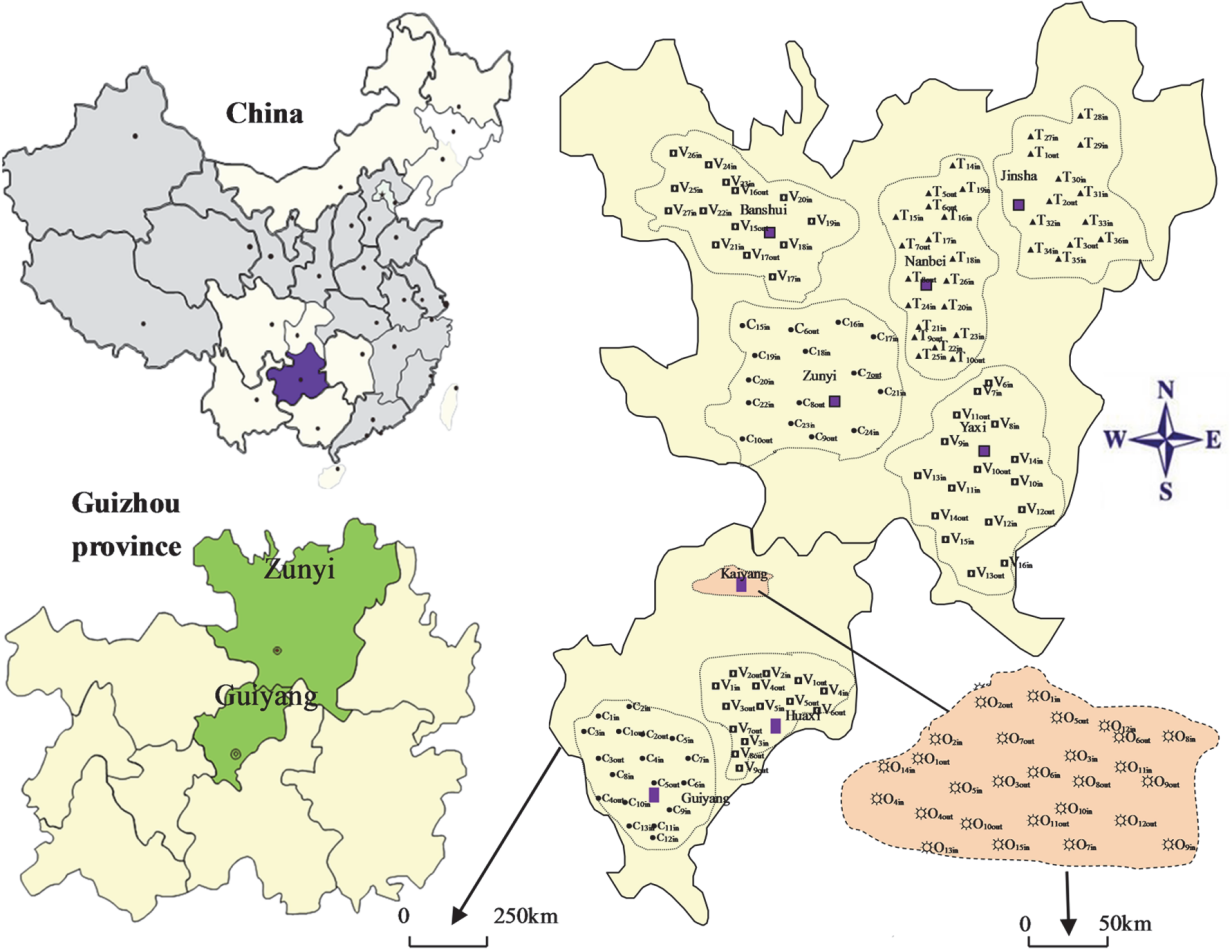
Geographical locality of the sample collection site.

Guizhou is one of the least developed provinces in China, and the imbalance of urban and rural economic development is obvious. In order to explore the variances of PAHs sources from different areas characterized by different pollution situations, 88 indoor dust samples were randomly collected from 2 representative cities, 2 towns, 3 villages and one orefield in Guizhou province during autumn, 2012 (Fig. 1). To analyze the possible emission sources for PAHs in indoor dust, 48 outdoor dust samples were collected in the house sampling site areas. Table 1 gives a descriptive profile of the sampling environments in details. All of the sampling sites have been authorized by Science and Technology Department of Guizhou province (STDG).

**Table 1:** Characteristics of sampling locations.

Sampling location	Type	Longitude and latitude	Vehicles	Main heating way	Main cooking methods	Sample number
**Guiyang**	The most developed city of Guizhou	N26°37′38.45″, E106°41′56.28″	≈ 700000	Electric heating	Electricity and coal gas	C_1in_-C_13in_, C_1out_-C_5out_
**Zunyi**	The second developed city of Guizhou	N27°41′49.85″, E106°55′15.77″	≈ 600000	Electric heating	Electricity and natural gas	C_15in_-C_24in_, C_6out_-C_10out_
**Nanbei**	Town	N27°32′27.88″, E106°49′50.35″	≈ 80000	Electric heating	Electricity and coal gas	T_14in_-T_26in_, T_5out_-T_10out_
**Jinsha**	Town	N27°26′46.22″, E106°15′53.77″	≈ 70000	Electric heating	Electricity and coal gas	T_27in_-T_36in_, T_1out_-T_3out_
**Huaxi**	Village	N26°23′42.97″, E106°39′18.71″	≈ 900000	Electric heating and coal firing	Electricity and coal	V_1in_-V_5in_, V_1out_-V_9out_
**Yaxi**	Village	N27°34′56.25″, E106°40′15.96″	≈ 30000	Electric heating and coal firing	Coal	V_6in_-V_16in_, V_10out_-V_14out_
**Banshui**	Village	N27°31′21.33″, E106°21′50.31″	≈ 20000	Electric heating and coal firing	Coal	V_17in_-V_27in_, V_15out_-V_17out_
**Kaiyang**	The largest phosphate orefiled in Guizhou	N27°04′20.74″, E107°02′10.63″	≈ 30000	Electric heating and coal firing	Electricity and coal	O_1in_-O_15in_, O_1out_-O_12out_

“C”, “T”, “V”, and “O” represent the city, town, village and orefield, respectively.

“in” and “out” represent the indoor and outdoor dust, respectively.

Indoor dust samples were all collected from the place above the floor level where dust accumulates easily, such as the surfaces of shelves, upholstery and door frames. To obtain an adequate dust sample for analysis, collection of multiple samples was necessary. For example, dust from several different rooms including bedroom, living room, hall and dining room where children are likely to stay in a residence may have been collected and composited into one sample. Outdoor dusts were collected from the surface of house balcony, outside windowsill. etc., and all of which were over 50 cm off the ground.

All dust samples were collected with a polyethylene brush. To prevent cross-contamination, brushes were cleaned between samples by ultrasonic rinsing in water for 5 min, rinsed with deionized water three times, and then air dried. The dust samples were brought to the laboratory and placed in a desiccator for 48 h, sieved through 80 μm screen, and finally oven dried at 45°C.

### Chemicals and materials

A composite standard solution of 18 PAHs including acenaphthene(ANA), acenaphthylene (ANY), benzo(b)fluoranthene (BbF), benzo(k)fluoranthene (BkF), benzo(g,h,i)perylene (BPE), chrysene (CHR), fluoranthene (FLT), fluorene (FLU), naphthalene (NAP), phenanthrene (PHE), anthracene (ANT), benzo(a)- anthracene (BEA), benzo(j)fluoranthene (BjF), benzo(a)pyrene (BaP), benzo(e)pyrene (BeP), bibenzo(a,h)anthracene (BaA), indeno(1,2,3-cd)pyrene (IPY) and pyrene (PYR) was purchased from Sigma-Aldrich (USA), each at a concentration of 1000 μg·mL^−1^. N-hexane and acetonitrile were obtained from Chongqing Xinyu Chemical Reagent Co., Ltd. (China), both with an analytical grade.

### Sample preparation and analysis

Dust samples were dried by a controllable temperature oven (45°C) and then crushed into powder by versatile grinder (XuLang co., LTD, Chengdu, China). Milled samples were kept in a constant temperature oven (Bosu co., LTD, Shanghai, China 25°C) to prevent deterioration. A 0.5 g aliquot of dust sample was added with 5 mL of dichloromethane and then placed into an ultrasonic cleaner (Jining ultrasonic equipment co., LTD, China). The mixture was extracted for 30 min and then the extraction solution was centrifugally separated (10000 r·min^−1^ for 10 min). The supernatant was separated and rotary evaporation concentrated to 1 mL, then added to chromatography column equipped with 1 g anhydrous sodium sulfate and 2 g silica for purification, with 8 mL n-hexane for prewash, again eluted with 10 mL of n-hexane—dichloromethane (1+1) mixture solution, collected n-hexane—dichloromethane solution and concentrated to dryness using a gentle stream of nitrogen. Dissolved the residue to 1 mL with methanol, filtered through 0.45 μm Millipore membrane and an aliquot of 10 μL of the filtrate was used for High Performance Liquid Chromatograph (HPLC) analysis.

The PAH analysis was conducted on an Agilent series of 1260 HPLC, equipped with a vacuum degasser, a quaternary pump, an auto sampler and a diode array detector system. Data collection was performed using *Chem.-Station* software (Agilent USA). ZORBAX Eclipse PAH column (2.1×100 mm, 1.8 μm) from Agilent was used with the mobile phase consisting of acetonitrile (A) and water (B). The optimized gradient elution was performed using the following linear gradient: 0 min—3 A%, 5 min—5 A%, 10 min—10 A%, 15 min—95 A%, 50 min—95 A%. The column compartment was kept at the temperature of 35°C and detection wavelength was 220 nm.

### Quality assurance/quality control

#### Calibration curves, limits of detection (LODs) and limits of quantification (LOQs)

The stock solution containing 18 markers was prepared and diluted to appropriate concentration ranges for the establishment of calibration curves. The calibration graphs were plotted after linear regression (Table 2) of the peak areas versus the corresponding concentrations. LODs and LOQs were determined at signal—to—noise ratios (S/N) of about 3 and 10, respectively. In the present study, LODs and LOQs of 18 PAHs were in the range of 6.18–12.58 ng·g^−1^ and 20.60–41.93 ng·g^−1^ dry weights, respectively.

**Table 2:** Linear regression data, LOD and LOQ of investigated compounds.

Analytes	Linear regression data			LOD (ng)	LOQ (ng)
	Regressive equation	γ	Linear range (μg)		
**Acenaphthene**	Y = 821.13X + 2.39	0.9998	0.20–1.99	12.42	41.40
**Acenaphthylene**	Y = 508.75X—31.22	0.9999	0.20–1.99	7.46	24.87
**Benzo(b)fluoranthene**	Y = 629.67X + 12.02	0.9997	0.20–2.00	7.53	25.10
**Benzo(k)fluoranthene**	Y = 322.18X—19.31	0.999 5	0.20–2.01	12.58	41.93
**Benzo(g,hi)perylene**	Y = 389.55X + 10.27	0.9998	0.20–1.99	12.45	41.50
**Chrysene**	Y = 263.42X—18.66	0.9997	0.20–1.99	7.42	24.73
**Fuoranthene**	Y = 375.94X + 8.25	0.9996	0.20–2.00	6.47	21.57
**Fluorene**	Y = 136.17X + 5.74	0.9999	0.20–2.00	12.49	41.63
**Naphthalene**	Y = 408.23X + 14.43	0.9993	0.20–1.99	10.96	36.53
**Phenanthrene**	Y = 399.35X + 16.64	0.9997	0.20–2.00	8.05	26.83
**Anthracene**	Y = 359.11X—12.47	0.9998	0.20–2.00	6.41	21.37
**Benzo(a)anthracene**	Y = 369.82X + 7.63	0.9996	0.20–1.99	6.18	20.60
**Benzo(j)fluoranthene**	Y = 321.16X + 2.52	0.9997	0.20–2.01	9.09	30.30
**Benzo(a)pyrene**	Y = 211.52X + 4.68	0.9993	0.20–2.00	8.02	26.73
**Benzo(e)pyrene**	Y = 188.29X—9.65	0.9995	0.20–1.99	7.93	26.43
**Dibenzo(a,h)anthracene**	Y = 177.32X + 5.45	0.9997	0.20–1.98	10.39	34.63
**Indeno(1,2,3-cd) pyrene**	Y = 210.36X + 10.12	0.999 9	0.20–1.99	9.32	31.07
**Pyrene**	Y = 155.21X—7.38	0.9996	0.20–2.00	10.69	35.63

All the analytes showed good linearity (γ > 0.999) in the concentration ranges.

In the linear regression data, Y refers to the peak area, X is the concentration, and γ is the correlation coefficient of the equation.

#### Precision, repeatability and stability

Precision was evaluated with both mixed standards solution and sample solution under the selected optimal conditions six times in 1 day for inter-day variation and twice a day on 3 consecutive days for intra-day variation. Repeatability was confirmed with six different working solutions prepared from sample C_1in_ and one of them was injected into the apparatus every 2 h within 12 h to evaluate the stability of the solution. All the results were expressed as relative standard deviations (RSD), and lower than 3%, which indicated that this examination method had a good precision, repeatability and stability.

#### Recovery and Robustness

The recovery was performed by adding known amount of the 18 standard substance and the spiked samples were then extracted, processed, and quantified in accordance with the methods mentioned above. The mean recoveries ranged from 91.67% to 102.81%. Method robustness was tested on ZORBAX Eclipse PAH C_18_ column (250 mm × 4.6 mm, 5 μm) and Waters PAH C_18_ column (250 mm × 4.6 mm, 5 μm). The same sample solution was separately analyzed and contents of the 18 characteristic constituents were calculated. Mean contents of the 18 compounds were 0.29, 0.35, 1.02, 0.16, 0.58, 2.13, 0.44, 1.12, 0.89, 0.67, 1.03, 0.53, 0.76, 0.81, 1.46, 0.92, 0.62 and 0.15 μg·g^−1^ for ZORBAX Eclipse PAH column and 0.30, 0.34, 1.01, 0.16, 0.57, 2.14, 0.43, 1.13, 0.88, 0.66, 1.02, 0.52, 0.76, 0.82, 1.45, 0.93, 0.61 and 0.15 μg·g^−1^ for Waters PAH C_18_ column. No significant difference existed between the results from the two columns by t-test (*P* > 0.05), which indicated that the developed method was capable of producing results with acceptable performance.

### Risk assessment

The incremental lifetime cancer risk (ILCR) was developed to quantitatively estimate the exposure risk for environmental PAHs based on the U.S. EPA standard models[17]. The following assumptions underlie the model applied in the present study: (a) Human beings are exposed to indoor dust through three main pathways: ingestion, inhalation and dermal contact with dust particles; (b) Intake rates and particle emission can be approximated by those developed for soil particles; (c) Some exposure parameters of people in the observed areas are similar to those of reference populations; (d) The total carcinogenic risk could be computed by summing the individual risks calculated for the three exposure ways; (e) The cancer risk is assessed based on exposure under a type of land use pattern over the entire lifetime. The following models were widely used to evaluate the ILCR in terms of ingestion, dermal contact and inhalation:

$${ILCRs}_{Inhalation}\;=\;\frac{CS\times ({CSF}_{Inhalation}\times \sqrt[{3}]{\left(\frac{BW}{70}\right)})\times {IR}_{Inhalation}\times EF\times ED}{BW\times AT\times PEF}$$ (1)

$${ILCRs}_{Dermal}\;=\;\frac{CS\times ({CSF}_{Dermal}\times \sqrt[{3}]{\left(\frac{BW}{70}\right)})\times SA\times AF\times ABS\times EF\times ED}{BW\times AT\times 10^{6}}$$ (2)

$${ILCRs}_{Ingestion}\;=\;\frac{CS\times ({CSF}_{Ingestion}\times \sqrt[{3}]{\left(\frac{BW}{70}\right)})\times {IR}_{Ingestion}\times EF\times ED}{BW\times AT\times 10^{6}}$$ (3)

where CSF is carcinogenic slope factor (mg·kg^−1^·day^−1^)^−1^, BW is body weight (kg), AT is the average life span (year), EF is the exposure frequency (day·year^−1^), ED is the exposure duration (year), IR_Inhalation_ is the inhalation rate (m^3^·day^−1^), IR_Ingestion_ is the soil intake rate (mg·day^−1^), SA is the dermal surface exposure (cm^2^), AF is the dermal adherence factor (mg·cm^−2^·h^−1^), ABS is the dermal adsorption fraction, and PEF is the particle emission factor (m^3^·kg^−1^). CSF_Ingestion_, CSF_Dermal_ and CSF_Inhalation_ of BaP were addressed as 7.3, 25, and 3.85 (mg·kg^−1^·day^−1^)^−1^, respectively, determined by the cancer-causing ability of BaP [18]. Other parameters referred in the model for children (1–6 years old) and adults (7–31 years old) were based on the Risk Assessment Guidance of U.S. EPA and related publications, shown in Table 3. CS (μg·kg^−1^) is the sum of converted PAHs concentrations based on toxic equivalents of BaP using the Toxic Equivalency Factor (TEF) listed in Table 4 [19].

**Table 3:** Parameters used in the incremental lifetime cancer risk assessment.

Exposure variable	Unit	Adult	Child
**Exposure frequency (EF)** [20]	day·year^−1^	180	180
**Exposure duration (ED)**[21]	year	24	6
**Body weight (BW)** [22]	kg	61.5	15
**Dust ingestion rate (IR_ingestion_)**[21]	mg·day^−1^	100	200
**Inhalation rate (IR_inhalation_)**[21]	m^3^·day^−1^	20	10
**Dermal adherence factor (AF)**[21]	mg·cm^−2^	0.07	0.2
**Dermal exposure area (SA)**[21]	cm^2^	5700	2800
**Particle emission factor (PEF)**[21]	m^3^·kg^−1^	1.36×10^9^	1.36×10^9^
**Dermal adsorption fraction (ABS)**[21]	Unitless	0.13	0.13
**Averaging life span (AT)** [23]	day	70×365 = 25,550	70×365 = 25,550

**Table 4:** Summary of measured PAHs in indoor dust of Guizhou (μg·g^−1^).

PAH	Aromatic ring	TEF^b^	Mean				Minimum				Maximum				Median			
			City	Town	Village	Orefield	City	Town	Village	Orefield	City	Town	Village	Orefield	City	Town	Village	Orefield
**ANA**	3	0.001	0.08	0.05	0.04	0.07	0.03	0.02	0.02	0.02	0.14	0.09	0.09	0.12	0.11	0.06	0.04	0.08
**ANY**	3	0.001	0.16	0.13	0.12	0.16	0.09	0.08	0.07	0.08	0.23	0.18	0.19	0.27	0.19	0.16	0.12	0.15
**BbF**	5	0.10	1.07	0.80	0.19	0.79	0.58	0.49	0.11	0.31	1.42	1.04	0.26	1.02	1.01	0.88	0.21	0.73
**BkF**	5	0.10	0.16	0.11	0.07	0.17	0.09	0.06	0.04	0.06	0.25	0.15	0.12	0.29	0.18	0.13	0.08	0.22
**BPE**	6	0.01	0.58	0.54	0.48	0.84	0.24	0.21	0.18	0.32	0.74	0.66	0.52	1.05	0.24	0.21	0.18	0.32
**CHR**	4	0.01	0.72	0.60	0.33	1.58	0.40	0.33	0.15	0.82	1.05	0.92	0.79	1.98	0.73	0.39	0.22	1.56
**FLT**	4	0.001	1.14	0.88	0.42	1.37	0.42	0.29	0.25	0.63	1.67	1.51	0.83	2.05	1.23	1.06	0.51	1.42
**BeP**	3	1.00	0.20	0.16	0.11	0.12	0.12	0.08	0.02	0.01	0.28	0.26	0.26	0.26	0.18	0.17	0.13	0.15
**NAP**	2	0.001	ND^a^	ND^a^	ND^a^	0.05	ND^a^	ND^a^	ND^a^	0.02	ND^a^	ND^a^	ND^a^	0.07	ND^a^	ND^a^	ND^a^	0.05
**PHE**	3	0.001	0.58	0.73	0.77	0.95	0.22	0.51	0.49	0.58	0.78	0.95	0.98	1.19	0.22	0.51	0.49	0.58
**ANT**	3	0.01	0.05	0.04	0.06	0.08	0.05	0.01	0.02	0.02	0.03	0.08	0.07	0.09	0.13	0.01	0.02	0.02
**BEA**	4	0.10	0.47	0.40	0.24	0.53	0.21	0.17	0.09	0.21	0.73	0.77	0.51	0.91	0.55	0.41	0.23	0.77
**FLU**	5	0.001	0.38	0.27	0.20	0.34	0.16	0.12	0.10	0.14	0.62	0.46	0.37	0.58	0.47	0.29	0.25	0.39
**BaP**	5	1.00	0.29	0.25	0.17	0.44	0.12	0.13	0.09	0.19	0.41	0.34	0.28	0.77	0.29	0.25	0.15	0.53
**BjF**	5	0.10	0.46	0.28	0.28	0.99	0.18	0.13	0.15	0.29	0.72	0.42	0.53	1.56	0.49	0.32	0.32	0.89
**BaA**	5	1.00	0.35	0.29	0.16	0.20	0.18	0.14	0.09	0.07	0.71	0.55	0.32	0.47	0.56	0.39	0.14	0.24
**IPY**	6	0.10	0.35	0.31	0.29	0.55	0.13	0.11	0.09	0.14	0.52	0.47	0.41	0.73	0.13	0.11	0.09	0.14
**PYR**	4	0.001	0.65	0.54	0.34	0.48	0.27	0.23	0.22	0.21	0.91	0.72	0.58	0.79	0.57	0.49	0.37	0.55
**LMW** ^**c**^			1.07	1.11	1.1	1.43	0.51	0.7	0.62	0.73	1.46	1.56	1.59	2.00	0.83	0.91	0.80	1.03
**HMW** ^**d**^			6.62	5.27	3.17	8.28	2.98	2.41	1.56	3.39	9.75	8.01	5.52	12.2	6.45	4.93	2.75	7.76
**Σ**_**18**_ **PAHs**			7.69	6.38	4.27	9.71	3.49	3.11	2.18	4.12	11.21	9.57	7.11	14.20	7.28	5.84	3.55	8.79
**HMW/Σ**_**18**_ **PAHs**			0.86	0.83	0.74	0.85	0.85	0.77	0.72	0.82	0.87	0.84	0.78	0.86	0.89	0.84	0.77	0.88

^a^ Under the detection limit.

^b^PAHs toxic equivalency factor with respect to BaP.

^c^ Low molecular weight PAHs (2–3 rings PAHs)

^d^ High molecular weight PAHs (4–6 rings PAHs).

## Results and Discussion

### Level of PAHs

**Fig 2:**
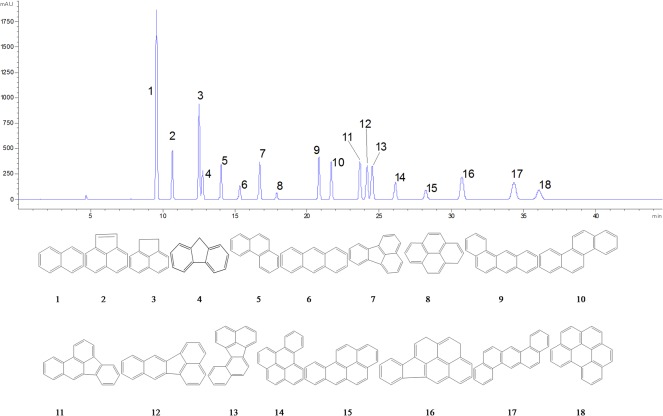
HPLC chromatograms and structures of 18 PAHs, 1–18: NAP, ANY, ANA, FLU, PHE, ANT, FLT, PYR, BaA, CHR, BbF, BkF, BjF, BeP, BaP, IPY, BEA and BPE.

Eighteen PAH compounds (Fig. 2) were detected in all dust samples, and the concentrations on dry weight basis of individual PAH in different areas were presented in Table 4. Concentrations of Σ_18_PAHs in dust of Guizhou province varied from 2.18 to 14.20 μg·g^−1^ with an average of 6.78 μg·g^−1^. This result indicated that PAHs tend to accumulate in dust particles, which could be used as an indicator of environmental pollution. The highest Σ_18_PAHs concentration was found in dust from orefield where the high concentrations of PAHs may have resulted from the emissions of mining activities and mineral processing operations [24].

PAH levels in indoor dust varied greatly in different functional areas (Table 4). The mean concentrations of Σ_18_PAHs decreased in the following order: City > town > village. The contributions of individual PAH in cities and towns are basically identical, and contribution rate of BbF, BPE, CHR, FLT, BEA, PHE, BjF and PYR are all over 5%. However, there is a significant difference to the contribution of individual PAH in village when comparing with city and town. BPE, FLT, BEA, BjF, PHE, PYR and IPY play the dominant role, their contribution rate are all over 5%. This finding was similar to the reports of PAHs distribution in the indoor dust of Palermo (Italy) and Sydney [25, 26].

In the specific sampling area, concentrations of Σ_18_PAHs increased in the following order (Table 5): Yaxi < Banshui < Huaxi < Nanbei < Jinsha < Zunyi < Guiyang < Kaiyang. This result was consistent with an earlier statement that mean concentrations of Σ_18_PAHs decreased in the following order: Orefield > City > town > village.

**Table 5:** Effect of functional type on individual PAH concentrations in dust samples of Guizhou (μg·g^−1^).

PAH	Guiyang	Huaxi	Kaiyang	Yaxi	Zunyi	Nanbei	Banshui	Jinsha
**ANA**	0.09	0.04	0.07	0.04	0.07	0.05	0.04	0.05
**ANY**	0.17	0.13	0.16	0.11	0.15	0.12	0.12	0.14
**BbF**	1.15	0.20	0.79	0.18	0.98	0.74	0.19	0.86
**BkF**	0.14	0.07	0.17	0.07	0.15	0.10	0.07	0.12
**BPE**	0.63	0.51	0.84	0.46	0.53	0.50	0.49	0.58
**CHR**	0.78	0.35	1.58	0.31	0.66	0.56	0.34	0.64
**FLT**	1.24	0.45	1.37	0.40	1.05	0.82	0.43	0.94
**BeP**	0.22	0.12	0.12	0.10	0.18	0.15	0.11	0.17
**NAP**	ND^a^	ND^a^	0.05	ND^a^	ND^a^	ND^a^	ND^a^	ND^a^
**PHE**	0.63	0.82	0.95	0.73	0.53	0.68	0.79	0.78
**ANT**	0.05	0.06	0.08	0.06	0.05	0.04	0.06	0.04
**BEA**	0.51	0.25	0.53	0.23	0.43	0.37	0.24	0.43
**FLU**	0.41	0.21	0.34	0.19	0.35	0.25	0.20	0.29
**BaP**	0.32	0.18	0.44	0.16	0.27	0.23	0.17	0.27
**BjF**	0.50	0.30	0.99	0.27	0.42	0.26	0.29	0.30
**BaA**	0.38	0.17	0.20	0.15	0.32	0.27	0.16	0.31
**IPY**	0.37	0.31	0.55	0.28	0.31	0.29	0.30	0.33
**PYR**	0.71	0.36	0.48	0.32	0.60	0.50	0.35	0.58
**Σ**_**18**_ **PAHs**	8.30	4.53	9.71	4.06	7.05	5.93	4.35	6.83

^a^ Under the detection limit.

In addition, mean concentration of Σ_18_PAHs in indoor dust was at least 10% higher than that of outdoors (Table 6). Specifically, mean concentrations of Σ_18_PAHs for indoor dust were 11.2%, 17.0%, 12.7% and 20.2% higher than that of outdoor in city, town, village and orefield, respectively. This tendency was same as the report of Kliucininkas [27], and indicated that special attention should be given to PAH pollution of indoor environment because of most people spending more than 80% of their time in indoor environment [28]. The mean level of Σ_18_PAHs in this study was relatively higher than those in the United Kingdom (0.002 μg·g^−1^), Norway (0.0069 μg·g^−1^), Canada (0.0011 μg·g^−1^), Australia (0.0033 μg·g^−1^) and Greater Cairo, Egypt (0.045–2.61μg·g^−1^) [29], but lower than those of Shanghai, China (21.44 μg·g^−1^), Birmingham, UK (12.56–93.70 μg·g^−1^) and Ulsan, Korea (11.8–245 μg·g^−1^) [30, 31].

**Table 6:** Comparison of measured mean PAHs in dust samples of Guizhou (μg·g^−1^).

PAH	Aromatic ring	City		Town		Village		Orefield	
		Indoor	Outdoor	Indoor	Outdoor	Indoor	Outdoor	Indoor	Outdoor
**ANA**	3	0.08	0.07	0.05	0.05	0.04	0.05	0.07	0.07
**ANY**	3	0.16	0.15	0.13	0.13	0.12	0.13	0.17	0.16
**BbF**	5	1.16	0.97	0.84	0.72	0.18	0.22	0.91	0.67
**BkF**	5	0.18	0.15	0.12	0.10	0.08	0.06	0.19	0.14
**BPE**	6	0.41	0.36	0.38	0.27	0.31	0.38	0.93	0.76
**CHR**	4	0.79	0.65	0.66	0.48	0.40	0.20	1.68	1.47
**FLT**	4	1.21	1.07	0.96	0.71	0.51	0.24	1.52	1.22
**BeP**	3	0.18	0.23	0.18	0.11	0.08	0.19	0.15	0.09
**NAP**	2	ND^a^	ND^a^	ND^a^	ND^a^	ND^a^	ND^a^	ND^a^	ND^a^
**PHE**	3	0.40	0.36	0.31	0.29	0.17	0.17	0.47	0.39
**ANT**	3	0.15	0.12	0.14	0.18	0.11	0.16	0.13	0.14
**BEA**	4	0.48	0.46	0.41	0.39	0.26	0.21	0.55	0.51
**FLU**	5	0.40	0.36	0.27	0.27	0.18	0.25	0.36	0.33
**BaP**	5	0.31	0.27	0.26	0.23	0.19	0.14	0.47	0.42
**BjF**	5	0.49	0.44	0.30	0.24	0.29	0.27	1.07	0.92
**BaA**	5	0.38	0.32	0.32	0.23	0.17	0.15	0.22	0.19
**IPY**	6	0.65	0.60	0.37	0.44	0.24	0.30	0.41	0.31
**PYR**	4	0.65	0.65	0.56	0.51	0.22	0.03	0.56	0.41
**LMW**		0.97	0.93	0.81	0.76	0.52	0.70	0.99	0.85
**HMW**		7.11	6.30	5.45	4.59	3.03	2.45	8.87	7.35
**Σ**_**18**_ **PAHs**		8.08	7.23	6.26	5.35	3.55	3.15	9.86	8.20

^a^ Under the detection limit.

### PAHs composition pattern

The 18 PAHs were grouped according to aromatic ring number: low molecular weight PAHs (LMW, 2–3 rings PAHs) and high molecular weight PAHs (HMW, 4–6 rings PAHs). HMW PAHs dominated in all sampling sites, and the average percentage of HMW PAHs to total PAHs was 82%, with a range of 72% to 89% (Table 4). HMW PAHs were mainly derived from high-temperature combustion process (such as vehicular exhaust, mining processing activities, etc.) and LMW PAHs were chiefly originated from low or moderate temperature combustion (such as coal burning) [32, 33]. Chinese transportation network and number of vehicles had grown explosively. During our field survey, cars could be seen almost everywhere, even the undeveloped rural areas. Combining the compositional pattern of PAHs by ring size, it was inferred that the PAHs in indoor dusts were probably dominated by vehicular exhaust. And the low or moderate temperature combustion such as coal burning also contributed a portion of the PAHs inputs for most sampling sites. The possible sources of PAHs would be further discussed with other evidences in following section.

### Concentration ratios of PAHs

Identifying the possible sources of PAHs is important in understanding the fate and transport of PAHs in house environment. Several PAHs isomeric ratios have been used to identify different sources that contribute PAHs to environmental samples [34]. For example, the isomeric ratios of ANT/(ANT+PHE), BEA/(BEA+CHR), FLT/(FLT+PYR) and IPY/(IPY+BPE) have been used to distinguish between petrogenic and pyrolytic sources [35]. The ratio of ANT/(ANT +PHE) <0.1 suggests a petroleum source, while a ratio >0.1 reflects combustion [36]. Meanwhile, FLT/(FLT +PYR) <0.4 indicates petroleum, between 0.4 and 0.5 implies liquid fossil fuel combustion, and a ratio >0.5 is the characteristic of biomass and coal combustion [37]. IPY/(IPY +BPE) and BEA/(BEA+CHR) may characterize the nature of potential PAH emission sources. That is, IPY/(IPY+BPE) <0.2 and BEA/(BEA+CHR) <0.2 are indications of petroleum and petrogenic sources. When BEA/(BEA+CHR) falls between 0.2 and 0.35 and IPY/(IPY+BPE) between 0.2 and 0.5, the PAHs usually come from petroleum combustion (liquid fossil fuel, vehicle and crude oil combustion). When IPY/(IPY+BPE) >0.5 and BEA/(BEA+CHR) >0.5, it strongly indicates the contribution of coal, grass and wood[38].

As shown in Table 7, ratios of FLT/(FLT +PYR) are generally above 0.5 while ANT/(ANT +PHE) lower than 0.1, suggesting a mixed source of coal combustion and traffic emission. This result agreed with the conclusion that vehicular traffic and coal combustion are major contributors of atmospheric PAHs in Guizhou province [39, 40] and was consistent with the sources of PAHs in urban surface dust in central Shanghai [41] and Guangzhou areas [42]. In addition, values of IPY/(IPY+BPE) ratio varied between 0.304 and 0.441 while BEA/(BEA+CHR) varied between 0.207 and 0.464, revealing vehicular traffic emissions as the main source. This supported the result from a previous study that vehicles were the dominant source of particulate PAHs in the cities of the Istanbul, Turkey [43].

**Table 7:** Isomeric ratios for indoor dust samples.

Ratio	City			Town			Village			Orefield		
	Min	Max	Mean	Min	Max	Mean	Min	Max	Mean	Min	Max	Mean
**ANT/(ANT+PHE)**	0.043	0.038	0.039	0.049	0.093	0.069	0.084	0.098	0.079	0.052	0.072	0.078
**BEA/(BEA+CHR)**	0.330	0.413	0.392	0.343	0.464	0.405	0.382	0.394	0.422	0.207	0.312	0.256
**FLT/(FLT+PYR)**	0.611	0.653	0.635	0.562	0.684	0.627	0.531	0.593	0.556	0.705	0.751	0.724
**IPY/(IPY+BPE)**	0.351	0.344	0.333	0.304	0.413	0.416	0.441	0.410	0.376	0.365	0.377	0.396

### PCA-MLR Analysis

Concentration ratios for PAHs could only provide qualitative information about the contribution of various sources. In order to enhance the accuracy of source identification, principal component analyses with multiple linear regression analysis (PCA-MLR) was used to conduct quantitative assessments. PCA-MLR model is a multivariate analytical tool widely used for receptor modeling in environmental source apportionment studies [44, 45].

**Fig 3:**
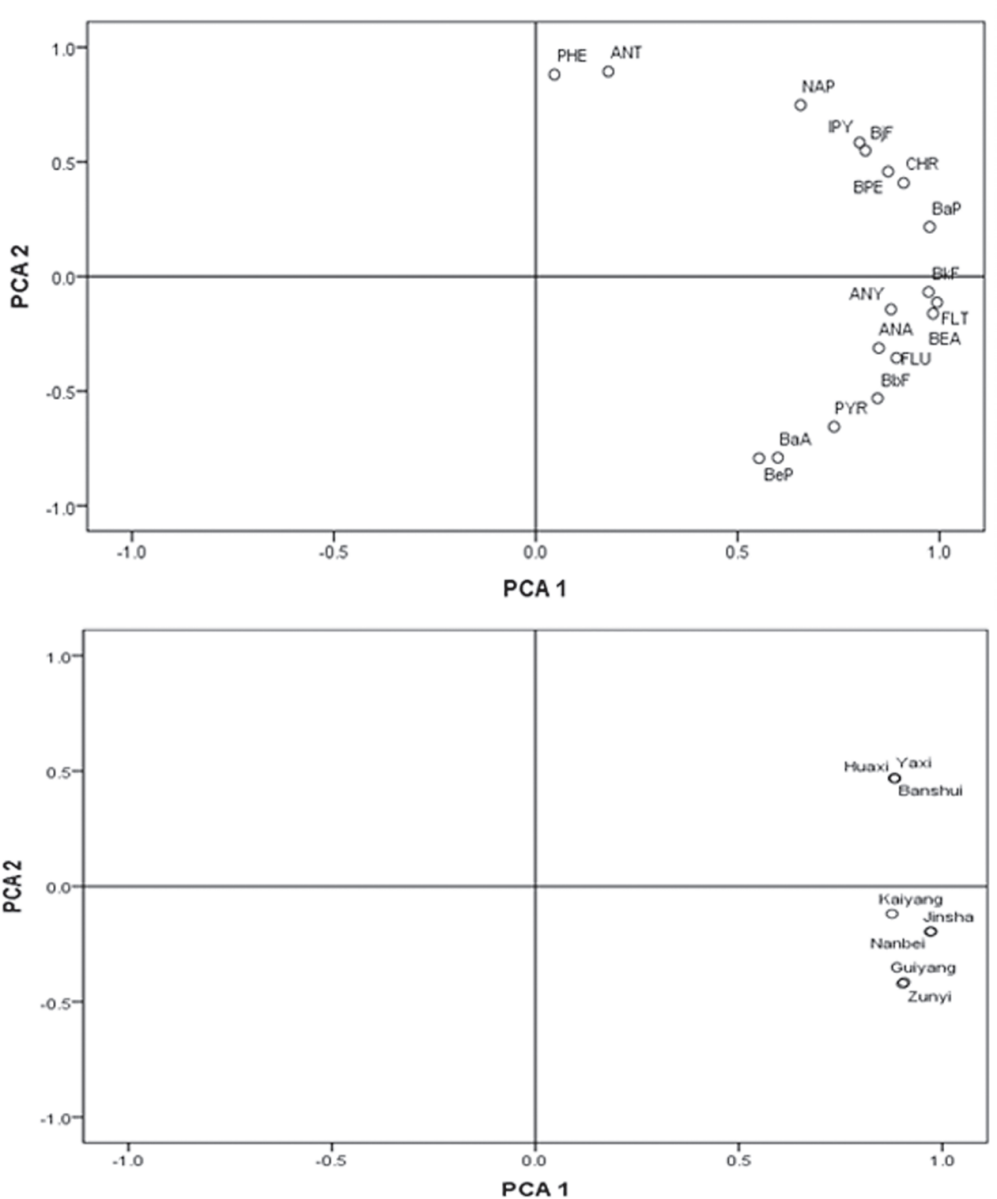
Plot with PC1 and PC2 from principal component analysis. (a) Factor loadings of 18 PAHs on two components, and (b) factor scores of sampling locations on the PC1 and PC2.

After varimax rotation, 2 factors (eigenvalue >1) were extracted by PCA (Fig. 3A). Factor 1 was responsible for 82.86% of the total variance. This factor got high loading for BeP, BaA, PYR, BbF, FLU, BEA, ANA, FLT, ANY and BkF. These species were mainly associated with the petroleum and transportation combustion emission [44, 46]. Thus this factor might be the vehicle exhaust source categories. Factor 2 (13.74% of the total variance) correlated with PHE, ANT, NAP, IPY, BjF, CHR, BPE and BaP, represented the source of diesel mission [47, 48].

Next, the contributions of these 2 factors (sources) were estimated by PCA-MLR model. As shown in Fig. 3B, the samples sites of Kaiyang, Jinsha, Nanbei, Guiyang and Zunyi were characterized by higher score of factor 1, while Huaxi, Yaxi and Banshui characterized by higher score of factor 2.

### Cluster analysis(HCA)

**Fig 4:**
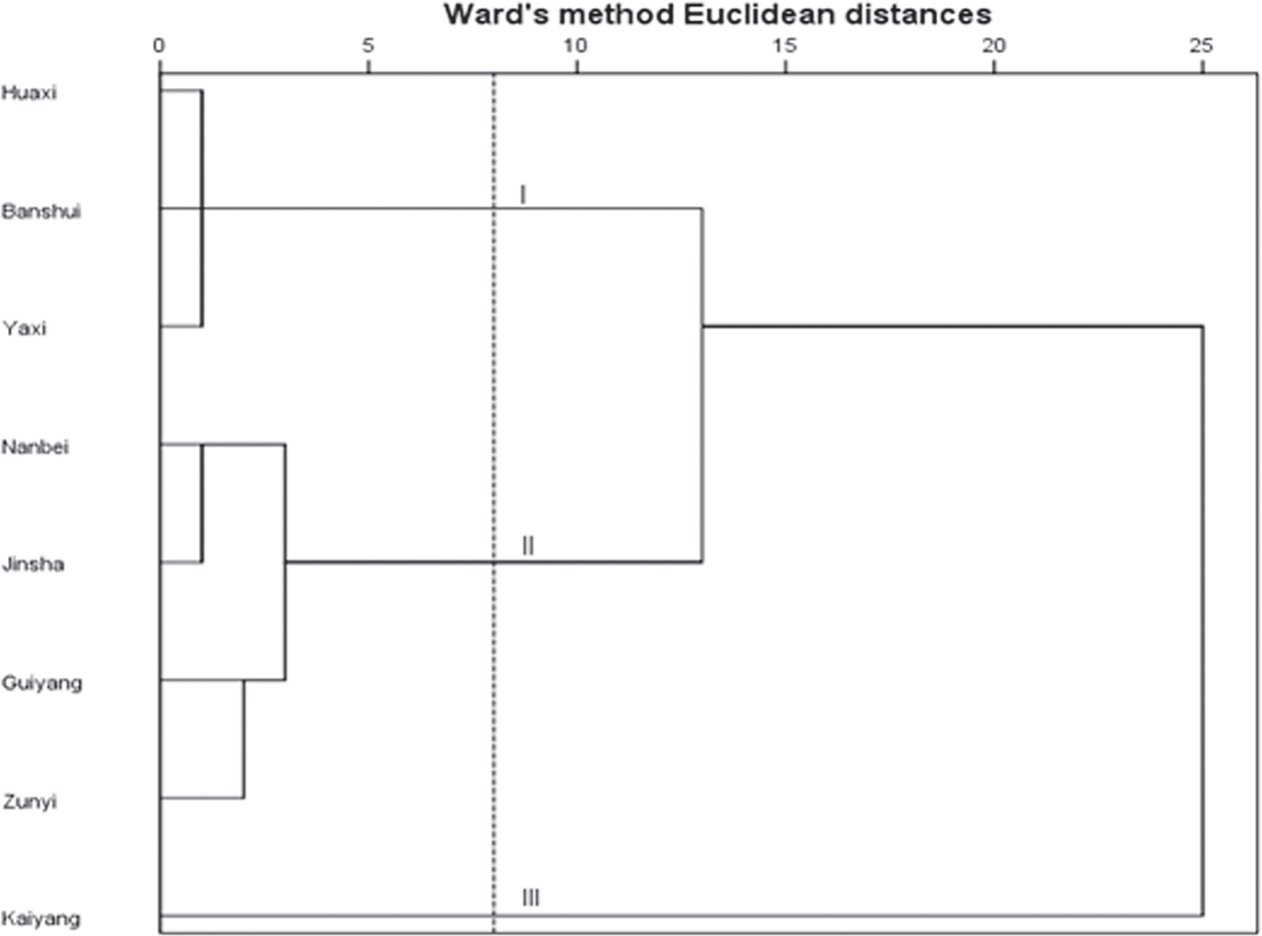
Clustering analysis diagram.

Cluster analysis or clustering is the task of grouping a set of objects in such a way that objects in the same group (called a cluster) are more similar (in some sense or another) to each other than to those in other groups (clusters) [49]. In the present study, PAHs levels of different sites were analyzed by HCA and the result was shown in Fig. 4. Three groups were discriminated.

Sites of Huaxi, Yaxi and Banshui were clustered in group 1. They were all located in the village of Guizhou province. These areas are dominated by agriculture and are sparsely populated. Cooking activity and a small amount of transportation were the main pollution sources in these regions. Sites of Nanbei, Jinsha, Guiyang and Zunyi were clustered in group 2. Guiyang and Zunyi were the most developed cities of Guizhou province, and Nanbei and Jinsha were the representative town in Guizhou. They have the common characteristics of dense population, prosperous business and well-developed transportation. Kaiyang was clustered in group 3, where had 78% high-quality phosphate rock resources of China and an accompanying boom in mining activities and other phosphate industry.

### Health risk assessment

The goal of this study was to evaluate the potential cancer risk of human exposure to indoor dust PAHs of Guizhou province. The ILCR was taken as an ensign to identify the age-specific potential cancer risks in the study of human exposure to environmental PAH pollution sources [50, 51]. Depending on the Toxic Equivalence Factor (TEF) and carcinogenic slope factor (CSF), a probabilistic risk assessment framework was applied to estimate risk incurred from exposure routes of inhalation, ingestion and dermal contact (Table 8). The cancer risk levels via dermal contact and ingestion pathway ranged from 10^−7^ to 10^−6^ in all the dust samples, while the mean cancer risk via inhalation was 10^−10^ to 10^−11^, about 10^3^ to 10^5^ times lower than that through ingestion and dermal contact. This was also observed in a study of exposure to PAHs in urban surface dust of Guangzhou[23]. Therefore, inhalation of re-suspended particles through mouth and nose was almost negligible, when compared with the other routes.

**Table 8:** The potential cancer risk for each sampling zone and exposure pathway.

Sampling zone	CS (μg·kg^−1^)	Child				Adult			
		Ingestion	Dermal contact	Inhalation	Cancer risk	Ingestion	Dermal contact	Inhalation	Cancer risk
**City (mean)**	1.11×10^3^	2.73×10^−6^	3.41×10^−6^	5.31×10^−11^	6.14×10^−6^	2.13×10^−6^	3.79×10^−6^	1.65×10^−10^	5.92×10^−6^
**City (min)**	5.47×10^2^	1.35×10^−6^	1.68×10^−6^	2.62×10^−11^	3.03×10^−6^	1.05×10^−6^	1.87×10^−6^	8.15×10^−11^	2.92×10^−6^
**City (max)**	1.79×10^3^	4.41×10^−6^	5.49×10^−6^	8.56×10^−11^	9.90×10^−6^	3.44×10^−6^	6.11×10^−6^	2.67×10^−10^	9.55×10^−6^
**Town (mean)**	9.04×10^2^	2.23×10^−6^	2.77×10^−6^	4.32×10^−11^	5.00×10^−6^	1.74×10^−6^	3.09×10^−6^	1.35×10^−10^	4.83×10^−6^
**Town (min)**	4.53×10^2^	1.12×10^−6^	1.39×10^−6^	2.17×10^−11^	2.51×10^−6^	8.71×10^−7^	1.55×10^−6^	6.75×10^−11^	2.42×10^−6^
**Town (max)**	1.46×10^3^	3.59×10^−6^	4.48×10^−6^	6.98×10^−11^	8.07×10^−6^	2.81×10^−6^	4.98×10^−6^	2.18×10^−11^	7.79×10^−6^
**Village (mean)**	5.58×10^2^	1.37×10^−6^	1.71×10^−6^	2.67×10^−11^	3.08×10^−6^	1.07×10^−6^	1.90×10^−6^	8.32×10^−11^	2.97×10^−6^
**Village (min)**	2.52×10^2^	6.20×10^−7^	7.73×10^−7^	1.20×10^−11^	1.39×10^−6^	4.84×10^−7^	8.60×10^−7^	3.76×10^−11^	1.34×10^−6^
**Village (max)**	1.06×10^3^	2.61×10^−6^	3.25×10^−6^	5.07×10^−11^	5.86×10^−6^	2.04×10^−6^	3.62×10^−6^	1.58×10^−10^	5.66×10^−6^
**Orefield (mean)**	1.09×10^3^	2.68×10^−6^	3.34×10^−6^	5.21×10^−11^	6.02×10^−6^	2.09×10^−6^	3.72×10^−6^	1.62×10^−10^	5.81×10^−6^
**Orefield (min)**	3.84×10^2^	9.45×10^−7^	1.18×10^−6^	1.84×10^−11^	2.12×10^−6^	7.38×10^−7^	1.31×10^−6^	5.72×10^−11^	2.05×10^−6^
**Orefield (max)**	1.99×10^3^	4.90×10^−6^	6.11×10^−6^	9.52×10^−11^	1.10×10^−5^	3.82×10^−6^	6.79×10^−6^	2.97×10^−10^	1.06×10^−5^

^a^ The sum of converted values of PAHs based on toxic equivalents of BaP using the Toxic Equivalency Factor (TEF).

In the case of children, the cancer risk levels via ingestion was within the same order of magnitude (10^−7^ to 10^−6^) as through dermal contact, indicating that both ingestion and dermal contact greatly contributed to the cancer risk for children. However, the risk value of direct ingestion for children was significantly higher (p<0.01) than the corresponding risk of ingestion for adults. The most sensitive subpopulation is young children because of their hand-to-mouth activity, whereby contaminated dust can be readily ingested [52]. In addition, with the lower body weight of children, the PAHs intake (mg per kg of body weight per day) of a child is believed to be greater than that of an adult. Furthermore, early development of organ, nervous, and immune systems could probably enhance the carcinogens sensitivity in children [50]. Thus, the hazard health risk for children exposed to urban dust PAHs are thought to be considerably greater than that of adults. Compared to children, the dermal contact appeared to be the predominant exposure route that induced a relatively higher risk for adults, followed by the ingestion pathway. Adult health risk due to dermal contact was significantly higher (p<0.01) than that for children. This finding was similar to the human cancer risk resulted from PAHs exposure in urban dust of Tianjing, China [18], which could be explained by the higher values of dermal exposure area (SA) and exposure duration (ED) of adults.

Under most regulatory programs, an ILCR between 10^−6^ and 10^−4^ indicated potential risk, where the virtual safety was denoted with an ILCR of 10^−6^ or less and a potentially high risk was estimated by an ILCR of greater than 10^−4^ [50]. In the present study, the ILCRs of cancer risk for both children and adults were higher than the base line value of acceptable risk, indicating a moderate potential carcinogenic risk. This showed that the risk due to indoor dust PAHs exposure was pervasive for residents in Guizhou province. The highest ILCR was found in the orefield, followed by the city and town, while the lowest in the village. The variation of ILCRs of cancer risk among the four functional areas indicated the great effects of PAH emission sources on health risk levels. Furthermore, it should not be overlooked that the high level of ILCR of 10^−5^ was found for adults and children in some places such as residential area near orefield where 440,000 people lived.

## Conclusions

A total of 136 dust samples (88 indoor and 48 outdoor) collected from Guizhou, southwest of China, were analyzed for 18 PAHs. The total PAHs concentrations ranged from 2.18 μg·g^−1^ to 14.20 μg·g^−1^ with the mean value of 6.78 μg·g^−1^. The level of PAHs in dust samples at study area showed that mining area was the most polluted region, followed by city, town and village. Indoor dust had a higher Σ_18_PAHs concentration than that of outdoor. The high molecular weight PAHs were the most predominant components. The total PAH concentrations in city and town were dependent on industrial emissions and vehicular exhausts, while those in village were mainly originated from cooking activity and a small amount of transportation. For mining areas, it might primarily derive from the mining activities and other phosphate industry. The mean Incremental Lifetime Cancer Risk (ILCR) due to human exposure to indoor dust PAHs in city, town, village and orefield of Guizhou province, China were 6.14×10^−6^, 5.00×10^−6^, 3.08×10^−6^, 6.02×10^−6^ for children and 5.92×10^−6^, 4.83×10^−6^, 2.97×10^−6^, 5.81×10^−6^ for adults, respectively. The results of this study would be helpful to understand the levels, distribution and sources of the PAHs in house dust, which can provide the information for improving living environment and human health in Guizhou province.
